# Sports Medicine and Movement Sciences

**DOI:** 10.1016/j.heliyon.2022.e08996

**Published:** 2022-02-23

**Authors:** Giuseppe Musumeci

**Affiliations:** a Department of Biomedical and Biotechnological Sciences, Human Anatomy and Histology Section, School of Medicine, University of Catania, Via S. Sofia 87, 95123, Catania, Italy; b Research Center on Motor Activities (CRAM), University of Catania, 95123, Catania, Italy; c Department of Biology, College of Science and Technology, Temple University, Philadelphia, PA, 19122, USA

Sports Medicine is a relatively new topic in medicine and includes a variety of medical and paramedical fields. Although sports medicine is mistakenly thought to be mainly for sports professionals/athletes, it actually encompasses the entire population, including the active and non-active healthy populations, as well as the sick [1]. Sports medicine also engages amateur sportsmen and strives to promote physical activity and quality of life in the general population. Hence, the field involves all ages from childhood to old age, aiming to preserve and support every person at every age. Sports medicine, which started developing in the 19^th^ century, is today a medical speciality.

Currently, there exist different technologies applied in the world of sports medicine dedicated to the detection of health problems. Evidence has demonstrated that virtual environments can be useful therapeutic tools with demonstrated positive outcomes. Modern technological advances have led to the implementation of digital devices, such as wearables and smartphones, which have been shown to provide opportunities for healthcare professionals and researchers to monitor physical activity and therefore engage patients in daily exercising. Additionally, the use of digital devices has emerged as a promising tool for improving frequent health data collection, disease monitoring, and supporting public health surveillance. The leveraging of digital data has laid the foundation for the development of a new concept of epidemiological study, known as “Digital Epidemiology”, which could contribute in the future to personalized and precision sports medicine.

The understanding of the importance of physical activity and fitness as part of a healthy lifestyle is increasing all over the world, as well as the number of amateur athletes and the profession of sports medicine takes a big part in this process.

Physical inactivity is the fourth leading cause of morbidity and mortality worldwide [2]. Regular physical activity is highly beneficial for the primary, secondary and tertiary management of many common chronic conditions. There is considerable evidence for the benefits of physical activity for cardiovascular disease, diabetes, obesity, musculoskeletal conditions, some cancers, mental health and dementia [3]. Yet there remains a large evidence-practice gap between physicians’ knowledge of the contribution of physical inactivity to chronic disease and routine effective assessment and prescription of physical activity.

The benefits of physical activity for the prevention and treatment of many chronic diseases are well established, including the infection of Sars-CoV-2. Considering the countless positive effects of exercise, planning an adapted physical activity in all phases of recovery (bed rest, rehabilitation, and post-hospitalization) of the patient represents an important strategy to mitigate the decline of cognitive functions and improve the physical and psychological wellbeing of subjects affected by COVID-19. Physical activity, if adapted to the needs of the individual, practiced consistently and regularly, shows a positive influence on the immune system due to its natural protective and anti-inflammatory action. Correct and constant physical exercise, even at home, at all ages and especially in the elderly, is an extra shield against Sars-CoV-2 [4]. Thanks to the Adapted Physical Activity patients improve the skills: psychological, mental, cardiorespiratory and muscular.

For some chronic conditions, structured exercise interventions are at least as effective as drug therapy. The adapted physical activity should be prescribed in the same way as pharmacological treatment, deciding on the “dosage” and “formulation” for each patient. The “dosage” is calculated to reach a specific level of efficacy that prevents or improves symptoms but does not result in toxic effects [3]. The exercise regime should always be "adapted" personalized and "tailored" since the level of exercise will depend on the tolerability of the individual, since the body of each of us always responds differently. No do-it-yourself or generalized training/protocols should be allowed, because physical activity if done poorly, can cause more damage than a sedentary lifestyle. As stated by the American College of Sports Medicine, physical activity should be prescribed/administered, alternatively or in association with drug treatment by the Sports and/or Family Physician and/or the Kinesiologist [5].

With sincere satisfaction and pride, I present to you the Special Issue titled *“Sports Medicine and Movement Sciences”*. This Special Issue bridging the gap between science and practice in the promotion of exercise and health and in the scientific assessment, study, and understanding of sports performance, sports injury prevention and treatment, exercise for health as non-surgical and non-pharmacological treatments, rehabilitation techniques, adapted physical activity, drugs in sport, and recommendations for training and nutrition.

This Special issue comprises 3 review articles and 16 original research publications from a number of Sports Medicine and Movement Sciences researchers [6, 7, 8, 9, 10, 11, 12, 13, 14, 15, 16, 17, 18, 19, 20, 21, 22, 23, 24]. Taken together, these articles are geared toward the advancement of our understanding Sports Medicine and Movement Sciences arena, including: Cognitive function; Brain health; Gait analysis; Biomechanics; Health sciences; Physiology; Physical activity; Occupational health; Musculoskeletal system; Evidence-based medicine; Aerobic threshold; Anaerobic threshold; Maximal oxygen uptake; Neuroscience; Exercise; Physical Activity; Balance; Metastability; Neuromuscular control; Prevention; Rehabilitation; Health Promotion; Anatomy; Health Technology; Three-dimensional motion analysis; Reliability; knee injury; Athletic pubalgia; Cardiology; Women's Health; Female athletes; Applied psychology; Clinical psychology; Paralympic sport; Goalball; Soccer; Cognitive psychology; Quality of life; Disability; Regenerative medicine; Osteoarthritis; Virtual reality; Sensorimotor control; Sports injury prevention; Epidemiology; Public health; Psychology; COVID-19; Pandemic; Quarantine; Home based exercise; IPAQ-SF; Psychological well-being; PGWBI; Nerve injury; Nerve regeneration; Therapeutic exercise; Wearable technologies; Sprint initiation; Step technique; Multi-directional movement; Novel training environments and digital devices; Adherence; Breast cancer; Lifestyle; Public Health and Digital Epidemiology.

I hope that readers of Heliyon enjoy reading these significant contributions that remind us of the crucial importance of interdisciplinary collaboration between those working in Sports Medicine and their counterparts in Movement Sciences.

## Conflict of interest declaration

The author of this editorial does not have any conflict of interests.
